# Improved unhealthy lifestyle habits in patients with high cardiovascular risk: results from a structured lifestyle programme in primary care

**DOI:** 10.1080/03009734.2019.1602088

**Published:** 2019-05-07

**Authors:** Lena Lönnberg, Elin Ekblom-Bak, Mattias Damberg

**Affiliations:** a Center for Clinical Research, County of Västmanland, Uppsala University, Västerås, Sweden;; b Department of Public Health and Caring Sciences, Family Medicine and Preventive Medicine, Uppsala University, Uppsala, Sweden;; c The Swedish School of Sports and Health Sciences, Stockholm, Sweden

**Keywords:** Cardiovascular prevention, general practice, hypertension, lifestyle habits, structured lifestyle programme, type 2 diabetes mellitus

## Abstract

**Background.** Physical activity, healthful dietary habits, and not smoking are associated with reduced cardiovascular morbidity and mortality. However, few studies have examined how counselling to improve poor lifestyle habits might be carried out in clinical practice. In Swedish primary care, structured lifestyle counselling is still not integrated into everyday clinical practice. The aim of the present study was two-fold: (1) to describe a novel lifestyle intervention programme in primary care; and (2) to evaluate change in unhealthy lifestyle habits over 1 year in men and women with high cardiovascular risk who participated in the lifestyle intervention programme.

**Method.** A single-group study with a 1-year follow-up was carried out. A total of 417 people was enrolled, median age 62 years (54% women), with either hypertension (69%), type 2 diabetes mellitus, or impaired glucose tolerance. The 1-year intervention included five counselling sessions that focused on lifestyle habits, delivered by a district nurse with postgraduate credits in diabetes care and the metabolic syndrome. All patients were offered in-depth counselling for one or more lifestyle habits when needed. Lifestyle habits were assessed by a questionnaire at baseline and 1-year follow-up. Total change was assessed using a nine-factor unhealthy lifestyle habit index.

**Results.** Favourable, significant changes were observed for physical activity, dietary habits, smoking, and stress over 1 year. Similar improvements were seen for both sexes and type of diagnosis.

**Conclusions.** The results support the utility of a multifactorial, structured approach to change unhealthy lifestyle habits for cardiovascular risk prevention in a primary care setting.

## Introduction

Heart attack and stroke are major killers in all parts of the world. About 80% of premature deaths from these causes could be avoided by controlling for the main risk factors such as physical inactivity, unhealthy diet, and tobacco use (1). International guidelines on cardiovascular disease (CVD) prevention include lifestyle counselling to improve unhealthy lifestyle habits with the aim of reducing cardiovascular risk (2). These guidelines emphasize that the highest clinical priority for prevention should be directed towards patients at high cardiovascular risk, such as those with type 2 diabetes mellitus (T2DM), impaired glucose tolerance (IGT), and hypertension (3). The prevalence of T2DM is 4%–5% in Sweden for people of all ages and increases with age up to 20% for people older than 70 years (4,5). There is a strong relationship between T2DM and overweight, especially abdominal obesity (4–6). The prevalence of hypertension is about 25% in Sweden and increases with advancing age; e.g. it is >60% in people aged 60 years and over (4,7). For prevention of future CVD, behavioural interventions, such as increased physical activity, weight reduction, and smoking cessation, are essential for treating both T2DM and hypertension (5,7).

Unhealthy lifestyle habits are common in the population. According to a national survey by the Public Health Agency of Sweden about lifestyle habits and living conditions in 2016, half of all women and two-thirds of all men have at least one unhealthy lifestyle habit (8). Smoking, alcohol overconsumption, physical inactivity, and poor dietary habits account for 20% of total health-care costs in Sweden (9). The recently published guidelines—National Guidelines for Prevention and Treatment of Unhealthy Lifestyle from the Swedish National Board of Health and Welfare—highlight the importance of health-care professionals in providing patients with the knowledge, tools, and support needed to improve their unhealthy lifestyle habits (10). However, scientific evaluations of programmes to improve lifestyle habits are scarce, despite the knowledge that healthy lifestyle habits are important for reducing cardiovascular risk (3,11–14). Structured lifestyle counselling is still not integrated into everyday clinical practice in primary care (15–17).

It is important to evaluate these programmes because of the lack of evaluation of structured lifestyle counselling programmes which use only the limited resources available at the primary care level. Thus, the aim of the present study is two-fold: (1) to describe a novel lifestyle intervention programme in primary care; and (2) to evaluate change in unhealthy lifestyle habits over 1 year in men and women with high cardiovascular risk that participated in the lifestyle intervention programme. We hypothesized that the structured lifestyle counselling programme would improve lifestyle habits in these patients with high cardiovascular risk.

## Methods

### Study design and population

We conducted a single-group study with a 1-year follow-up with before and after measurements. All data were collected consecutively and were registered in a database by one of five district nurses. People registered at Citypraktiken, a primary care unit in Västerås, Sweden, were enrolled during a 5-year period between October 2009 and September 2014. The inclusion criteria were age 18–75 years and for the first time meeting the diagnosis criteria of either hypertension (blood pressure >140/90 mm Hg), T2DM (fasting blood glucose level >7 mmol/L), or IGT (two-hour glucose levels of 7.8–11.0 mmol/L on the 75-g oral glucose tolerance test). Antihypertensive or cholesterol-lowering medication was prescribed when needed according to hypertension and diabetes guidelines. People with dementia or severe psychiatric disease were excluded (Table 1).

**Table 1.** Baseline characteristics of the study population (*n* = 316).

	Total, *n* = 316	Men, *n* = 146	Women, *n* = 170	T2DM + IGT, *n* = 99	Hypertension, *n* = 217
Sex (% women)	54%			40%	60%
Diagnosis				31%	69%
Age, years	62.0 (54.0–66.0)	62.0 (54.8–66.0)	62.0 (53.8–67.0)	63.0 (58.0–67.0)	61.0 (53.0–66.0)
Height, cm	170.0 (164.0–179.0)	180.0 (175.0–183.2)	165.0 (161.0–168.5)	172.5 (165.0–180.0)	170.0 (164.0–177.5)
Weight, kg	83.8 (73.0–96.0)	92.0 (82.8–104.0)	76.4 (68.0–87.0)	93.0 (80.8–108.2)	80.0 (71.8–92.3)
Body mass index, kg/m^2^	28.0 (25.6–32.0)	28.2 (26.0–31.9)	28.0 (25.0–32.7)	31.0 (27.2–35.8)	27.5 (25.0–31.0)
Waist circumference, cm	100.9 (92.0–109.0)	103.0 (98.0–112.0)	94.0 (87.4–105)	106.8 (97.6–115.5)	97.0 (89.5–103.5)
Blood pressure, mmHg	150/90 (140–160/80–95)	150/90 (140–160/80–95)	150/90 (140–160/80–95)	140/80 (130–150/75–90)	155/90 (140–165/85–100)
Predicted maximal oxygen uptake, mL O_2_/kg/min^a^	22.0 (18.0–25.5)	22.0 (17.0–26.0)	22.0 (18.0–25.0)	20.0 (15.3–23.0)	22.9 (18.4–26.0)
Total cholesterol, mmol	5.9 (5.2–6.7)	5.6 (4.8–6.6)	6.1 (5.5–6.9)	5.4 (4.6–6.5)	6.1 (5.5–6.8)
Low-density lipoprotein, mmol	3.8 (3.2–4.6)	3.8 (3.0–4.5)	3.9 (3.3–4.7)	3.4 (2.7–4.4)	4.0 (3.4–4.7)
High-density lipoprotein, mmol	1.3 (1.1–1.6)	1.2 (1.0–1.4)	1.5 (1.3–1.8)	1.2 (1.0–1.4)	1.4 (1.2–1.7)
Triglycerides, mmol	1.4 (1.0–2.0)	1.5 (1.1–2.2)	1.3 (1.0–1.7)	1.7 (1.3–2.2)	1.2 (0.9–1.7)
Fasting blood glucose, mmol	5.5 (5.0–6.6)	5.9 (5.1–7.2)	5.3 (4.9–6.1)	7.7 (6.5–9.1)	5.2 (4.7–5.7)
Metabolic syndrome	51%	60%	43%	77%	39%
Previous cardiovascular disease	6%	8%	4%	15%	2%
Antihypertensive medication	56%	60%	54%	66%	52%
Cholesterol-lowering medication	15%	21%	10%	36%	6%

Continuous data are presented as median (Q1–Q3).

a 269 individuals performed a bicycle ergometer test.

**Figure 1.**
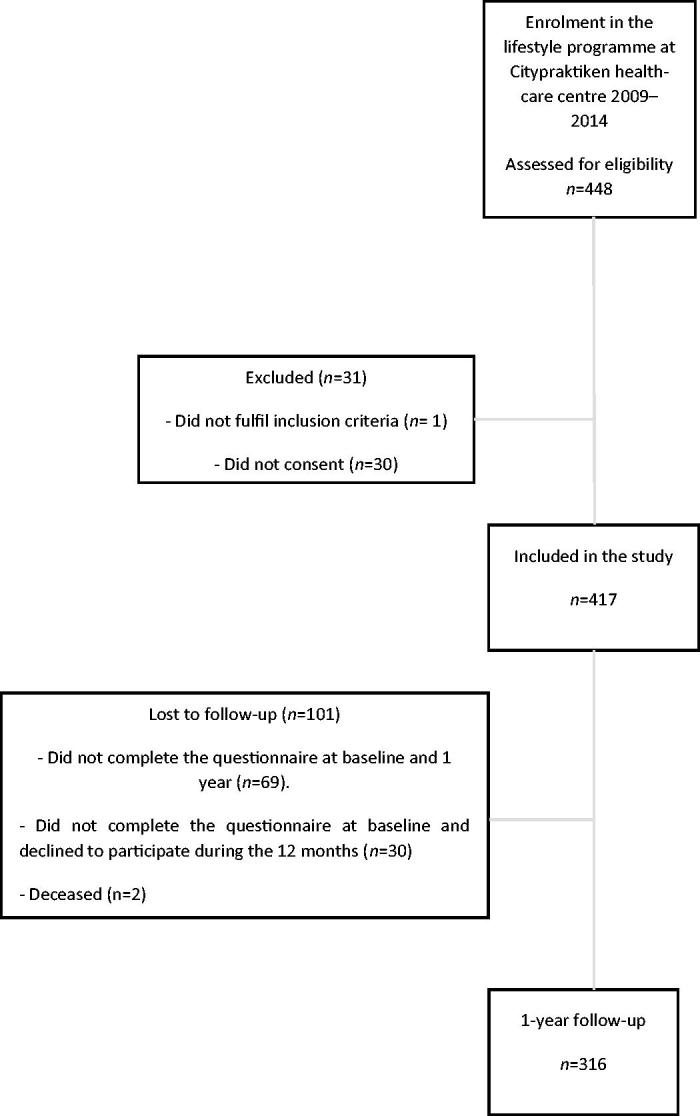
Flow chart.

A total of 448 people was referred by their physician to join the lifestyle programme. One did not meet the inclusion criteria, and 30 did not provide written consent. Thus, 417 participants were included in the present study. However, 101 were lost to follow-up: 69 did not complete the questionnaire at the baseline and 1-year follow-up, 30 did not complete the programme, and two died (Figure 1).

The study was conducted according to the ethical guidelines of the Declaration of Helsinki and the Good Clinical Practice guidelines. The Swedish Ethical Review Authority approved the study (reference number: 2014/497). All participants provided written informed consent. The study was registered at www.clinical-trials.gov (ClinicalTrial.gov ID: DNR 2014/497).

### The structured lifestyle programme

**Figure 2.**
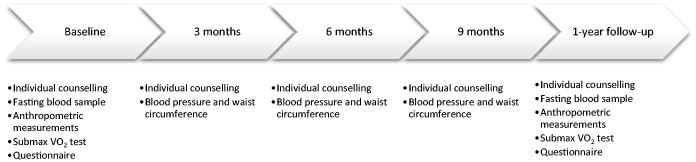
The structured lifestyle programme.

The structured lifestyle programme comprised five appointments with the same district nurse, with postgraduate credits in diabetes care and the metabolic syndrome, at the baseline and after 3, 6, 9, and 12 months. Fasting blood samples were obtained 1 week before, and a submaximal cycle ergometer test was performed the same day as the baseline and 1-year follow-up appointments. Anthropometric variables were measured, and the questionnaire was completed at baseline and the 1-year follow-up appointment. Blood pressure and waist circumference were measured at every appointment (Figure 2).

At both the baseline and 1-year follow-up appointment, the results of the clinical examination, anthropometric measurements, answers from the questionnaire, and laboratory data were discussed between the nurse and participant. Every appointment focused primarily on lifestyle habits and involved motivational interviewing to strengthen the participant’s ability to modify one or more lifestyle habits. Each participant received a prescription for physical activity in accordance with Professional Associations for Physical Activity, Physical Activity in the Prevention and Treatment of Disease, FYSS 2008 (17,18).

Dietary counselling was performed in accordance with the Nordic nutrition recommendations (19). If the participant needed extended counselling about one or more lifestyle habits, he or she was referred within the primary care unit, e.g. to a physiotherapist regarding physical activity or to a nurse trained in smoking cessation counselling. At the 1-year follow-up appointment, the nurse summarized the past 12-month period and new goals were set for the future. An oral referral response was given to the referring physician.

During the 12-month intervention, all participants were allowed to participate in three evening group sessions, alone or together with a spouse or a friend. The group session focused on: (1) cardiovascular risk factors and physical activity; (2) healthy food and alcohol and tobacco use; and (3) stress, sleeping habits, and behavioural change. The participation rate varied over time, and between 15% and 25% of all participants attended one or more of the group sessions.

### Clinical examination

At the baseline and 1-year follow-up, all participants were weighed in light indoor clothing without shoes to the nearest 0.1 kg using an electronic balance. Height was measured without shoes to the nearest 0.5 cm using a scale fixed to the wall. Body mass index was calculated from the measured weight and height as kg/m^2^. Waist circumference was measured with the participant in a standing position midway between the lower rib margin and the iliac crest with a tape measure to the nearest 0.5 cm. Blood pressure was measured using the standard auscultatory method with an appropriate-sized cuff on the right arm with the participant in a seated position after a 10-min rest. Maximal oxygen uptake was estimated using the Åstrand submaximal cycle ergometer test (20,21) on a Monark E 818 or Monark E 928 cycle ergometer.

### Laboratory measurements

The concentrations of total cholesterol (mmol/L), low-density lipoprotein (LDL, mmol/L), high-density lipoprotein (HDL, mmol/L), and triglycerides (mmol/L) were analysed by standard methods at Aleris MediLab (Stockholm, Sweden). The laboratory was quality-certified according to ISO/IEC 151 89. Fasting blood glucose concentration was measured by the laboratory at the primary care unit. All equipment was calibrated regularly.

### Questionnaire

Information about lifestyle habits was obtained using a self-administered questionnaire comprising 25 questions covering physical activity, dietary habits, alcohol consumption, tobacco use, stress, and sleeping habits. The questionnaire combined validated and reliability tested questions from other questionnaires regarding lifestyle habits. However, the complete questionnaire is not validated and tested for reliability in its present form. Questions about physical activity covered daily activity, exercise, and time being sedentary using a 1–4 scale and overall physical activity using a 0–10 visual analogue scale. Dietary habits were assessed in eight questions about the intake of fish, fruit and vegetables, fast food, extra calories, and soft drinks. Alcohol consumption was assessed in two questions about the frequency and quantity. Smoking and snuff use were assessed in questions covering daily consumption (yes/no) and number of cigarettes/portions of snuff. Stress and sleep habits were evaluated in two questions using a 1–4 scale. The questions have been listed in Tables 2–4. For the full questionnaire, see appendix.

**Table 2.** Self-reported physical activity-related lifestyle habits.

	Total			Sex-specific				Diagnosis-specific			
Continuous lifestyle habit questionnaire scoring	Baseline mean (SD)	1 year mean (SD)	*P* value over 1 year		Baseline mean (SD)	1 year mean (SD)	*P* value over 1 year		Baseline mean (SD)	1 year mean (SD)	*P* value over 1 year
Daily physical activity (*n* = 315)	3.13 (0.80)	3.25 (0.72)	0.001^a^	Men (*n* = 146)	2.95 (0.82)	3.14 (0.70)	<0.001^a^	T2DM + IGT (*n* = 99)	3.14 (0.71)	3.22 (0.68)	n.s.
				Women (*n* = 169)	3.29 (0.74)	3.34 (0.73)	n.s.	HT (*n* = 216)	3.13 (0.83)	3.26 (0.74)	0.002^a^
Exercise (*n* = 313)	2.23 (1.10)	2.71 (1.03)	<0.001^a^	Men (*n* = 146)	2.27 (1.12)	2.71 (1.04)	<0.001^a^	T2DM + IGT (*n* = 98)	2.18 (1.12)	2.73 (1.01)	<0.001^a^
				Women (*n* = 167)	2.20 (1.08)	2.71 (1.02)	<0.001	HT (*n* = 215)	2.26 (1.10)	2.70 (1.04)	<0.001^a^
Overall physical	4.45 (1.79)	5.92 (1.70)	<0.001^a^	Men (*n* = 144)	4.54 (1.90)	5.92 (1.62)	<0.001^a^	T2DM + IGT (*n* = 96)	4.35 (1.78)	5.99 (1.67)	<0.001^a^
activity (*n* = 312)				Women (*n* = 168)	4.37 (1.70)	5.93 (1.77)	<0.001^a^	HT (*n* = 216)	4.50 (1.80)	5.90 (1.71)	<0.001^a^
Time sedentary (*n* = 312)	1.86 (0.72)	1.79 (0.67)	0.015^a^	Men (*n* = 143)	1.93 (0.73)	1.87 (0.68)	n.s.	T2DM + IGT (*n* = 95)	1.95 (0.72)	1.86 (0.69)	n.s.
				Women (*n* = 169)	1.79 (0.71)	1.72 (0.66)	n.s.	HT (*n* = 217)	1.82 (0.72)	1.76 (0.66)	n.s.
% with unhealthy lifestyle habits	Baseline	1 year	Diff (95% CI)		Baseline	1 year	Diff (95% CI)		Baseline	1 year	Diff (95% CI)
Low daily physical activity level	19.4%	13.7%	−5.7% (−9.6 to −2.0)	Men	26.7%	15.8%	−10.9% (−17.6 to −4.5)	T2DM + IGT	19.2%	12.1%	−7.1% (−14.8 to 0.3)
(<30 min/day)				Women	13.0%	11.8%	−1.2% (−5.7 to 3.2)	HT	19.4%	14.4%	−5.0% (−9.7 to −0.7)
Low exercise level	56.5%	36.4%	−20.1% (−25.3 to −14.7)	Men	57.5%	41.1%	−16.4% (−24.1 to −8.4)	T2DM + IGT	58.2%	34.7%	−23.5% (−33.1 to −12.9)
(<1 hour/week)				Women	55.7%	32.3%	−23.4% (−30.3 to −15.9)	HT	55.8%	37.2%	−18.6% (−24.7 to −12.2)
Low overall physical activity	33.3%	9.0%	−24.3% (−29.6 to −19.2)	Men	36.8%	6.9%	−29.9% (−37.9 to −21.7)	T2DM + IGT	32.3%	7.3%	−25.0% (−34.9 to −15.1)
(≤3 on a 10-point scale)				Women	30.4%	10.7%	−19.6% (−26.5 to −12.9)	HT	33.8%	9.7%	−24.1% (−30.3 to −17.9)
High amount of sedentary time	16.0%	12.8%	−3.2% (−6.8 to 0.3)	Men	18.9%	17.5%	−1.4% (−7.2 to 4.4)	T2DM + IGT	18.9%	15.8%	−3.1% (−10.1 to 3.6)
(≥9 hours/day)				Women	13.6%	8.9%	−4.7% (−9.6 to −0.2)	HT	14.7%	11.5%	−3.2% (−7.7 to 1.1)

Daily physical activity was assessed with the question, ‘How physically active are you during a day?’ The response options were: 1, not at all physically active; 2, <30 min/day; 3, 30–60 min/day; 4, >60 min/day. Exercise was assessed using the question, ‘How much exercise do you perform in a week’. The response options were: 1, no activity at all; 2, <1 hour weekly; 3, 1–2 hours weekly; 4, >2 hours weekly. Overall physical activity was assessed by asking participants, ‘Please rate your overall physical activity from 0 to 10, with 0 indicating not at all physically active and 10 indicating very physically active’. Sedentary time was assessed by asking participants, ‘Please estimate the amount of time you sit each day’. The response options were: 1, 0–4 hours/day; 2, 5–8 hours/day; 3, 9–12 hours/day; 4, 13 hours a day or more.

a Significant change over 1 year, *P* value <0.05 with Bonferroni–Holm correction.

HT: hypertension; IGT: impaired glucose tolerance; n.s.: non-significant change; T2DM: type 2 diabetes mellitus.

**Table 3.** Self-reported dietary-related lifestyle habits.

	Total			Sex-specific				Diagnosis-specific			
Continuous lifestyle habit questionnaire scoring	Baseline mean (SD)	1 year mean (SD)	*P* value over 1 year		Baseline mean (SD)	1 year mean (SD)	*P* value over 1 year		Baseline mean (SD)	1 year mean (SD)	*P* value over 1 year
Fish (*n* = 316)	3.00 (0.78)	3.12 (0.76)	<0.001^a^	Men (*n* = 146)	2.94 (0.78)	3.07 (0.73)	0.009^a^	T2DM + IGT (*n* = 99)	3.11 (0.71)	3.14 (0.74)	n.s.
				Women (*n* = 170)	3.06 (0.77)	3.16 (0.78)	0.017^a^	HT (*n* = 217)	2.95 (0.80)	3.11 (0.76)	<0.001^a^
Fast food (*n* = 316)	2.27 (0.87)	2.19 (0.87)	0.036^a^	Men (*n* = 146)	2.41 (0.88)	2.34 (0.83)	n.s.	T2DM + IGT (*n* = 99)	2.40 (0.87)	2.36 (0.90)	n.s.
				Women (*n* = 170)	2.15 (0.85)	2.06 (0.89)	n.s.	HT (*n* = 217)	2.21 (0.87)	2.11 (0.85)	0.027^a^
Fruit and vegetables (*n* = 316)	3.63 (0.88)	3.80 (0.82)	<0.001^a^	Men (*n* = 146)	3.40 (0.88)	3.66 (0.66)	<0.001^a^	T2DM + IGT (*n* = 99)	3.61 (0.74)	3.80 (0.52)	0.001^a^
				Women (*n* = 170)	3.84 (0.48)	3.91 (0.32)	n.s.	HT (*n* = 217)	3.65 (0.72)	3.80 (0.52)	<0.001^a^
Extra calories (*n* = 316)	2.14 (0.88)	2.03 (0.82)	0.003^a^	Men (*n* = 146)	2.16 (0.90)	2.10 (0.88)	n.s.	T2DM + IGT (*n* = 99)	1.43 (0.64)	1.34 (0.54)	n.s.
				Women (*n* = 170)	2.12 (0.86)	1.96 (0.75)	0.003^a^	HT (*n* = 217)	1.43 (0.58)	1.39 (0.57)	0.023^a^
Soft drinks/juice (*n* = 316)	1.43 (0.60)	1.38 (0.56)	0.041^a^	Men (*n* = 146)	1.59 (0.65)	1.51 (0.62)	n.s.	T2DM + IGT (*n* = 99)	1.43 (0.64)	1.34 (0.54)	n.s.
				Women (*n* = 170)	1.29 (0.52)	1.26 (0.46)	n.s.	HT (*n* = 217)	1.43 (0.58)	1.39 (0.57)	n.s.
% of participants with unhealthy lifestyle habits	Baseline	1 year	Diff (95% CI)		Baseline	1 year	Diff (95% CI)		Baseline	1 year	Diff (95% CI)
Low intake of fish	4.7%	2.5%	−2.2% (−4.7 to −0.2)	Men	5.5%	2.1%	−3.4% (−7.9 to 0.0)	T2DM + IGT	3.0%	2.0%	−1.0% (−6.4 to 4.1)
(rarely or never)				Women	4.1%	2.9%	−1.2% (−4.6 to 1.9)	HT	5.5%	2.8%	−2.7% (−5.9 to −0.3)
High intake of fast food (≥ a	8.5%	5.7%	−2.8% (−6.2 to 0.4)	Men	11.0%	6.2%	−4.8% (−10.6 to 0.7)	T2DM + IGT	12.1%	8.1%	−4.0% (−11.8 to 3.4)
couple of times a month)				Women	6.5%	5.3%	−1.2% (−5.5 to 3.0)	HT	6.9%	4.6%	−2.3%| (−6.1 to 1.2)
Low intake of fruit and	9.8%	4.1%	−5.7% (−8.9 to −3.0)	Men	17.1%	7.5%	−9.6% (−15.2 to −4.8)	T2DM + IGT	11.1%	5.1%	−6.0% (−12.5 to −0.8)
vegetable (≥3 times a week)				Women	3.5%	1.2%	−2.3% (−6.3 to 1.0)	HT	9.2%	3.7%	−5.5% (−9.6 to −2.2)
High intake of extra	8.2%	6.0%	−2.2% (−5.1 to 0.5)	Men	10.3%	8.9%	−1.4% (−5.9 to 3.0)	T2DM + IGT	6.1%	3.0%	−3.1% (−8.7 to 1.5)
calories (daily)				Women	6.5%	3.5%	−3.0% (−7.3 to 0.9)	HT	9.2%	7.4%	−1.8% (−5.7 to 1.8)
High intake of soft	3.8%	2.5%	−1.3% (−3.6 to 0.8)	Men	6.2%	4.1%	−2.1% (−6.3 to 1.6)	T2DM + IGT	4.0%	1.0%	−3.0% (−8.8 to 1.6)
drinks/juice (daily)				Women	1.8%	1.2%	−0.6% (−3.8 to 2.4)	HT	3.7%	3.2%	−0.5% (−3.3 to 2.2)

The amount of fish consumed was assessed with the question, ‘How often do you eat fish?’ The response options were: 1, rarely/never; 2, a couple of times a month; 3, once a week; 4, a couple of times a week or more. The amount of fast food was assessed with the question, ‘How often do you eat sausages, hamburger, or pizza?’ The response options were: 1, rarely/never; 2, a couple of times a month; 3, once a week; 4, a couple of times a week or more. The amount of fruit and vegetables was assessed with the question, ‘How often do you eat fruit and vegetables?’ The response options were: 1, a few times a month; 2, 1–2 times a week; 3, 3–5 times a week; 4, daily. Consumption of extra calories was assessed with the question, ‘How often do you eat “extra” calories?’ The response options were: 1, a few times a month; 2, 1–2 times a week; 3, 3–5 times a week; 4, daily. Consumption of soft drinks/juice was assessed with the question, ‘How often do you drink sweetened soft drinks or juice?’ The response options were: 1, a few times a month; 2, 1–2 times a week; 3, 3–5 times a week; 4, daily.

a Significant change over 1 year, *P* value <0.05 with Bonferroni–Holm correction.

HT: hypertension; IGT: impaired glucose tolerance; n.s. = non-significant change over 1 year; T2DM: type 2 diabetes mellitus.

**Table 4.** Self-reported levels of alcohol consumption, tobacco use, stress, and sleeping difficulties.

	Total			Sex-specific				Diagnosis-specific			
Continuous lifestyle habit questionnaire scoring	Baseline mean (SD)	1 year mean (SD)	*P* value over 1 year		Baseline mean (SD)	1 year mean (SD)	*P* value over 1 year		Baseline mean (SD)	1 year mean (SD)	*P* value over 1 year
Alcohol, frequency (*n* = 315)	2.52 (1.05)	2.48 (1.03)	n.s.	Men (*n* = 145)	2.74 (1.03)	2.69 (1.03)	n.s.	T2DM + IGT (*n* = 99)	2.46 (0.98)	2.38 (0.96)	n.s.
				Women (*n* = 170)	2.33 (1.02)	2.30 (1.00)	n.s.	HT (*n* = 216)	2.54 (1.08)	2.52 (1.06)	n.s.
Alcohol, intake/occasion	0.99 (0.46)	0.97 (0.44)	n.s.	Men (*n* = 145)	1.09 (0.55)	1.06 (0.52)	n.s.	T2DM + IGT (*n* = 99)	0.99 (0.48)	0.97 (0.46)	n.s.
(*n* = 315)				Women (*n* = 170)	0.91 (0.34)	0.89 (0.33)	n.s.	HT (*n* = 216)	1.00 (0.45)	0.97 (0.42)	n.s.
Stress (*n* = 315)	2.82 (0.79)	2.73 (0.8)	0.009^a^	Men (*n* = 145)	2.59 (0.80)	2.58 (0.82)	n.s.	T2DM + IGT (*n* = 98)	2.58 (0.80)	2.58 (0.84)	n.s.
				Women (*n* = 170)	3.02 (0.73)	2.86 (0.78)	0.001^a^	HT (*n* = 217)	2.93 (0.77)	2.80 (0.80)	0.001^a^
Sleeping difficulties (*n* = 315)	2.59 (0.97)	2.54 (0.89)	n.s.	Men (*n* = 145)	2.32 (0.90)	2.33 (0.86)	n.s.	T2DM + IGT (*n* = 98)	2.55 (0.90)	2.57 (0.81)	n.s.
				Women (*n* = 170)	2.82 (0.96)	2.71 (0.87)	n.s.	HT (*n* = 217)	2.61 (1.01)	2.52 (0.92)	n.s.
% with unhealthy lifestyle habits	Baseline	1 year	Diff (95% CI)		Baseline	1 year	Diff (95% CI)		Baseline	1 year	Diff (95% CI)
Daily smoker	8.6%	6.0%	−2.6% (−5.1 to −0.3)	Men	9.0%	6.2%	−2.8% (−6.7 to 0.6)	T2DM + IGT	7.1%	7.1%	0.0% (−4.7 to 4.7)
				Women	8.2%	5.9%	−2.3% (−6.4 to 1.3)	HT	9.2%	5.5%	−3.7% (−7.2 to −0.7)
Snuff user	8.3%	8.3%	0.0% (−1.7 to 1.7)	Men	15.4%	16.1%	0.7% (−2.5 to 4.0)	T2DM + IGT	10.3%	10.3%	0.0% (−4.6 to 4.6)
				Women	2.4%	1.8%	−0.6% (−3.4 to 1.9)	HT	7.4%	7.4%	0.0% (−2.2 to 2.2)
Alcohol, high frequency (≥4	1.3%	1.6%	0.3% (−1.0 to 1.9)	Men	2.1%	2.8%	0.7% (−2.2 to 4.0)	T2DM + IGT	0.0%	0.0%	0.0% (−3.4 to 3.4)
times a week)				Women	0.6%	0.6%	0.0% (−2.5 to 2.5)	HT	1.9%	2.3%	0.4% (−1.5 to 2.7)
Alcohol, high intake per	9.5%	7.3%	−2.2% (−4.7 to 0.0)	Men	18.6%	15.2%	−3.4% (−8.1 to 0.9)	T2DM + IGT	11.1%	9.1%	−2.0% (−7.5 to 3.0)
occasion (≥5 glasses)				Women	1.8%	0.6%	−1.2% (−4.4 to 1.4)	HT	8.8%	6.5%	−2.3% (−5.4 to 0.4)
High level of stress (often)	19.0%	17.1%	−1.9% (−5.9 to 2.1)	Men	11.0%	13.1%	2.1% (−2.8 to 7.2)	T2DM + IGT	11.2%	12.2%	1.0% (−6.3 to 8.4)
				Women	25.9%	20.6%	−5.3% (−11.6 to 1.0)	HT	22.6%	19.4%	−3.2% (−8.2 to 1.7)
Sleeping difficulties (often)	19.7%	13.0%	−6.7% (−10.5 to −3.0)	Men	9.0%	8.3%	−0.7% (−5.1 to 3.6)	T2DM + IGT	16.3%	12.2%	−4.1% (−11.7 to 3.3)
				Women	28.8%	17.1%	−11.7% (−17.8 to −5.8)	HT	21.2%	13.4%	−7.8% (−12.4 to−3.6)

Alcohol frequency was assessed with the question, ‘How often do you drink alcoholic beverages?’ The response options were: 1, never; 2, less than once a month; 3, 2–4 times a month; 4, 1–3 times a week; 5, ≥4 times/week. Alcohol consumption was assessed with the question, ‘How many glasses do you typically drink when you drink alcohol?’ The response options were: 0, none; 1, 1–4 glasses; 2, 5–9 glasses; 3, ≥10 glasses. Daily smoking was assessed with the question, ‘Do you smoke? yes/no’. The use of snuff was assessed with the question, ‘Do you use snuff? yes/no’. The amount of stress was assessed with the question, ‘Do you feel stressed?’ The response options were: 1, never; 2, rarely; 3, sometimes; 4, often. Sleeping difficulties were assessed with the question, ‘Have you experienced difficulties with your sleep?’ The response options were: 1, never; 2, rarely; 3, sometimes; 4, often.

^a^Significant change over 1 year, *P* value < 0.05 with Bonferroni–Holm correction.

HT: hypertension; IGT: impaired glucose tolerance; n.s. = non-significant change; T2DM: type 2 diabetes mellitus.

### Unhealthy lifestyle habits and the unhealthy lifestyle habit index

Each continuous lifestyle habit variable was dichotomized further into unhealthy or healthy, considering the lowest rank on each question as unhealthy except for fruit and vegetable consumption where the two lowest ranks were considered as unhealthy (see Tables 2–4). To study the clustering of unhealthy lifestyle habits, a nine-factor unhealthy lifestyle habit index was constructed. This index included daily smoking, high alcohol intake, low daily physical activity, low exercise level, high sedentary time, low intake of fruit and vegetables, high fast-food consumption, sleeping difficulties, and high level of stress.

### The Care Need Index

The Care Need Index (CNI) is used to evaluate a population’s need for primary care. The CNI measures socio-economic factors and comprises seven variables; age >65 years; born in Eastern Europe, Asia, Africa, or South America; unemployed; single parent with child younger than 17 years; children under 5 years; low educational level; and highly mobile people. A high index indicates an increased need for health care (22).

### The metabolic syndrome and previous cardiovascular disease

The metabolic syndrome was classified according to the National Cholesterol Education Program (NCEP) Adult Treatment Panel III (ATPIII) (23). The NCEP/ATPIII has defined the metabolic syndrome as three or more of the following: waist circumference >102 cm in men and >88 cm in women; blood pressure ≥130/85 mmHg; triglyceride concentration ≥1.7 mmol/L; HDL concentration <1.0 mmol/L in men and <1.3 mmol/L in women; and fasting plasma glucose concentration >6.1 mmol/L. Individuals with antihypertensive or cholesterol-lowering medication were included in the high blood pressure or triglyceride groups.

Previous cardiovascular disease was defined as myocardial infarction, coronary insufficiency, angina, ischemic stroke, haemorrhagic stroke, transient ischaemic attack, peripheral artery disease, or heart failure.

### Statistics

Subgroup analyses were based on diagnosis set by the referring physician—individuals with hypertension, or individuals with either T2DM or IGT (T2DM + IGT)—and on sex. Continuous characteristic data were checked for a normal distribution. For questionnaire data, values from baseline were carried forward for missing data for all variables. Although the questionnaire responses were ordinal data, the mean and standard deviation values are presented to facilitate the interpretation of the results. However, to detect significant changes within groups over the year, the Wilcoxon signed-rank test was used. The Bonferroni–Holm correction was applied to reduce the possibility of getting a statistically significant result (i.e. type I error) when multiple hypothesis tests were performed. McNemar’s test was used to identify changes in percentages of each unhealthy lifestyle habits over the year. A paired-sample *t* test was used to detect changes in the unhealthy lifestyle index over the year. The analyses were performed using Statistical Package for Social Science (IBM SPSS Statistics for Windows, version 24.0, Armonk, NY, USA).

## Results

### Baseline characteristics

The population comprised 54% women, with a median age of 62 years (range 54–66 years), recently diagnosed with either hypertension (69%), type 2 diabetes mellitus (29%), or impaired glucose tolerance (2%) (Table 1). Patients lost to follow-up were significantly younger (*P* < 0.001), median age of 56 years (range 50–63.5 years), and had cholesterol-lowering medication to a lesser extent than the study population (5% for lost to follow-up compared to 15% in the study population, *P* = 0.006) (Table 1). Regarding weight, body mass index, and prevalence of metabolic syndrome, there were no differences between patients lost to follow-up and the study population. The Care Need Index (CNI) was 0.86 for the present primary care unit in 2014 compared with 1.08 (±0.20) in the county.

### Changes in physical activity and sedentary time

In the total sample, continuous levels of daily activity, exercise, and overall physical activity increased, and sedentary time decreased over the year (all *P* < 0.05) (Table 2). The percentages of participants with unhealthy (low) levels of daily activity, exercise, and overall physical activity decreased over the year.

Sex-specific analyses revealed that all activity-related habits improved in men, except that there was no significant improvement of sedentary time. By contrast, women showed a slight decrease in sedentary time and improved exercise and overall physical activity habits. Similar significant trends were seen for changes in the percentages of participants with the dichotomized unhealthy physical activity habits.

In participants with hypertension, daily activity, exercise, and overall physical activity increased over the year. There were similar significant improvements in the percentages of participants with low levels of these activity variables. In participants with T2DM, continuous scoring and the percentages of participants with low levels of exercise and overall activity improved over the year.

### Change in dietary habits

Continuous scoring of the intakes of fish, fast food, fruit and vegetables, extra calories, and soft drinks improved significantly over the year in the total sample (all *P* < 0.05) (Table 3). The percentages of participants with unhealthy low intakes of fish and fruit and vegetables decreased.

Sex-specific analyses revealed that both men and women increased their intake of fish. Men increased intake of fruit and vegetables, and women reduced their intake of extra calories (all *P* < 0.05). Men had a higher percentage of participants with unhealthy dietary habits than women at both the baseline and 1-year follow-up.

Participants with hypertension increased their intakes of fish and fruit and vegetables, and reduced their intakes of fast food and extra calories (all *P* < 0.05). In participants with T2DM, continuous intakes of fruit and vegetables increased (*P* < 0.001). The percentages of participants with unhealthy intake of fruit and vegetables decreased for both the T2DM and hypertension groups.

### Changes in alcohol consumption, tobacco use, stress, and sleeping habits

The number of daily smokers decreased in the total sample and for individuals with hypertension. Levels of stress decreased in the total sample, for women and individuals with hypertension respectively (all *P* < 0.01). The percentages of participants with unhealthy levels of stress and sleeping difficulties were nearly twice as high for women compared with men at both the baseline and 1-year follow-up. The percentages reporting sleeping difficulties decreased over the year in participants with hypertension (*P* < 0.05). A higher percentage of participants with hypertension had unhealthy levels of stress at the baseline.

### Changes in the unhealthy lifestyle habit index

**Figure 3.**
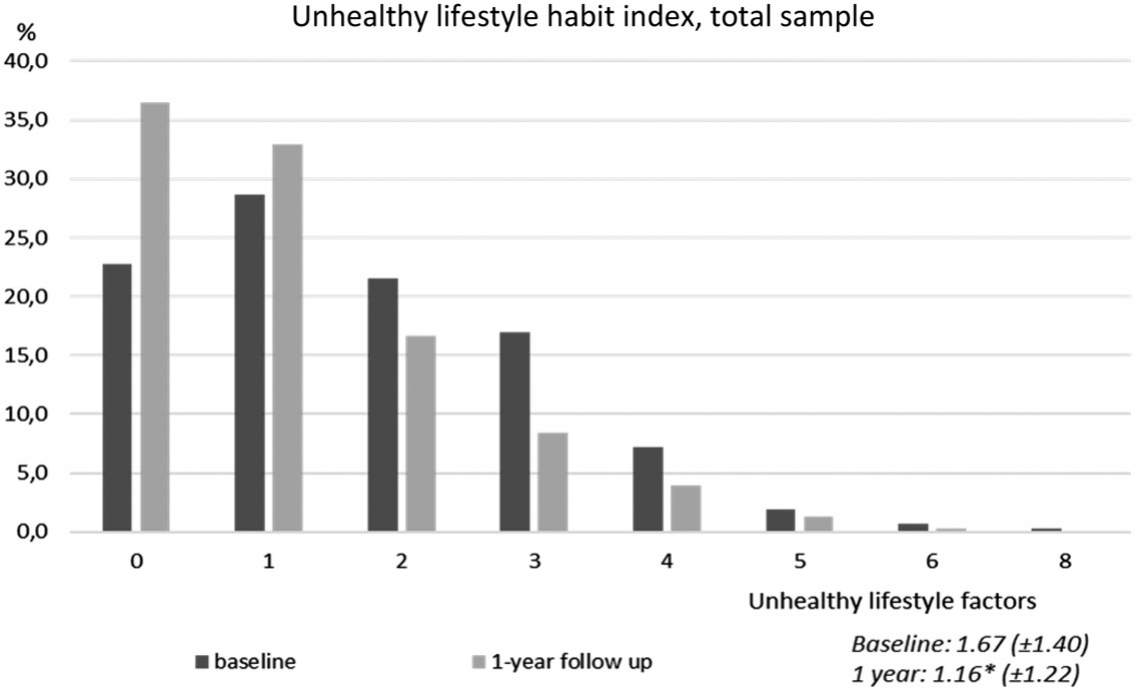
Unhealthy lifestyle habit index using nine factors, total sample. Data are expressed as the percentages of participants in the total sample. Mean value (SD) for number of unhealthy risk factors at baseline and 1-year follow-up (*P ≤ 0.001).

**Figure 4.**
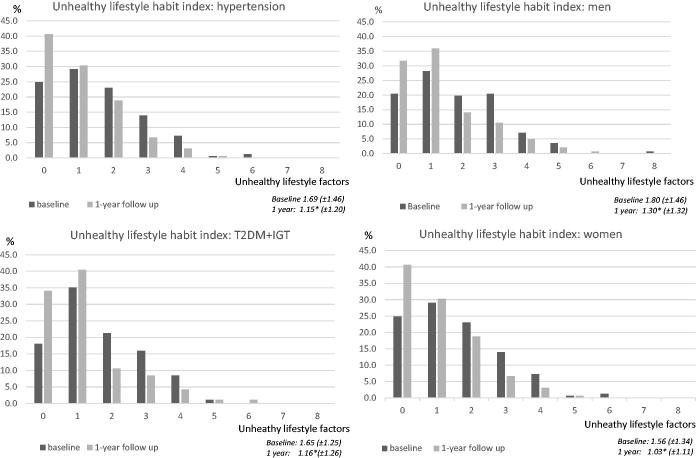
Unhealthy lifestyle habit index using nine factors, sex- and diagnosis-specific. Data are expressed as the percentages of participants in the total sample. Mean value (SD) for number of unhealthy risk factors at baseline and 1-year follow-up (*P ≤ 0.001). (IGT: impaired glucose tolerance; T2DM: type 2 diabetes mellitus).

The mean value of the unhealthy lifestyle habit index decreased over the year from 1.67 (±1.40) at the baseline to 1.16 (±1.22) at the 1-year follow-up (*P* < 0.001). At the end of the year, the percentage of the total sample with one or no unhealthy lifestyle habits had increased, and the percentages of those with two to eight risk factors had decreased (*P* < 0.001) (Figure 3). Men had a higher mean index at both the baseline (1.80 [±1.46]) and 1-year follow-up (1.30 [±1.32]) compared with women (1.56 [±1.34] and 1.03 [±1.11], respectively). The mean index decreased for both men and women over the year (*P* < 0.001). The mean index also decreased in both the hypertension and T2DM groups over the year, i.e. from 1.69 (±1.46) at the baseline to 1.15 (±1.20) at the 1-year follow-up (*P* < 0.001) in those with hypertension and from 1.65 (±1.25) to 1.16 (±1.26), respectively (*P* < 0.001), for those with T2DM (Figure 4).

## Discussion

The main findings of this study are the significant, favourable changes in physical activity levels, dietary habits, smoking, and feelings of stress and sleeping difficulties after participation in a 1-year structured lifestyle programme in people at high cardiovascular risk provided at a primary care unit. This was seen in both men and women, and in participants with hypertension or T2DM. To our knowledge, this is the largest Swedish study to evaluate a structured lifestyle programme performed in an ordinary clinical setting using only the limited resources available at a primary care centre. In people at high cardiovascular risk, changing lifestyle habits after participating in lifestyle counselling has been reported by Eriksson et al., whose study included 151 participants (16), and by Lidin et al., whose study included 100 participants (13).

In this study, the percentages of participants with one or no unhealthy lifestyle habits increased over the year from 51.5% to 69.4% (*P* < 0.001). This finding is important because having few unhealthy lifestyle habits is associated with reduced risk of incident ischemic CVD and all-cause mortality. Clustering of unhealthy lifestyle habits is, in our study, evaluated by a nine-factor unhealthy lifestyle habits index. It combines information about physical activity, diet, alcohol, smoking, stress, and sleeping difficulties. In 2013, Carlsson et al. could detect a 70% reduction for all-cause mortality in individuals with the healthiest lifestyle, using a seven-factor lifestyle habit index, in a representative population-based study of 4232 sixty-year-old men and women (24). Since the questions in our study’s questionnaire is not identical to the index used by Carlsson et al., we are not able to compare our results; however, a positive trend for all-cause mortality could be found in our study population as well.

There are different views on whether it is effective to address more than one lifestyle habit at the same time (4, 10). However, a structured lifestyle programme addressing one or more lifestyle habits has been shown to be successful in CVD prevention in other parts of Europe and in Sweden (12,13,16). A study by Gibson et al. that included 521 people with increased CVD risk reported significant favourable improvements in physical activity, dietary habits, and smoking cessation after a 16-week programme to improve unhealthy lifestyle habits (25). The structured lifestyle programme in our study provided people at high cardiovascular risk with the knowledge and tools to improve their unhealthy lifestyle habits, as stated in several guidelines to decrease the risk for future CVD (5,7,10). Our results are consistent with previous research evidence that individual counselling and support from a specialized nurse lead to improved lifestyle habits (13,16,25). The technique of motivational interviewing was chosen to provide person-centred care and makes it possible for individual participants to change one or more lifestyle habits. We regard this method as being both well-suited for clinical practice and feasible for implementing in primary care.

The increased physical activity level is important because even small increases in moderate-intensity physical activity provide health benefits (11,26). In our study, men reported a slightly higher level of both exercise and sedentary time than women, but a reverse relationship was observed for daily activity. This movement pattern is consistent with a Swedish cohort study of 948 Swedish men and women aged 50–64 years by Ekblom-Bak et al. (27).

The total sample showed changes in dietary habits to a healthier pattern. Similar results have been reported in both national and international studies (13,16,25). This is encouraging because following a healthy diet is associated with a reduced risk of CVD (28,29). In our study, men had a less healthy dietary pattern, which is consistent with other Swedish reports on dietary habits (30,31). Sex, low income, and low educational level are factors that should be considered when providing dietary counselling (10).

One-third of daily smokers stopped smoking after participating in the programme. This result is consistent with other reports of a 30%–40% success rate when participants are offered qualified counselling for smoking cessation, compared with 2%–3% when not given any support (31).

A concern that needs to be addressed is what the natural course of lifestyle changes would be for individuals after being diagnosed with either hypertension or T2DM. A study from Canada comprising 1281 persons newly diagnosed with hypertension showed that one of five persons quit smoking but that it did not lead to lasting lifestyle changes for e.g. physical activity, weight control, and alcohol use over a period of 2 years (33). Lifestyle change after a diagnosis of T2DM shows a similar pattern, with minimal changes in lifestyle factors after receiving a diagnosis, except for smoking cessation which was more common among persons with T2DM compared with those with no T2DM diagnosis according to an Australian study of persons newly diagnosed with T2DM by Chong et al. (34). This supports the possibility that the structured lifestyle programme might have contributed to the lifestyle changes seen in our study rather than the natural cause of lifestyle changes after being diagnosed.

The strength of this relatively large study is that the number of included participants has made subgroup analyses possible. It also provides a rather high external validity since the structured lifestyle programme was performed at an ordinary primary care unit.

Although the results achieved in this study are promising, there are several limitations. The study was not designed as a randomized controlled trial, and hence we cannot compare the changes in lifestyle habits in responses to this programme with those in standard care. This also means that regression towards the mean should be considered when interpreting the results.

The results must be seen in the context of a primary care unit with low CNI, indicating a population with a low proportion of individuals who are manual workers, unemployed, or foreign-born from non-Westerns countries (22). This can possibly limit the transference of the results of the structured lifestyle programme to primary care units with higher CNI.

The study involves the testing of many separate null hypotheses, which may entail problems with inflated type I error rates. To address this potential misinterpretation, we have undertaken a more stringent criterion for statistical significance level by using the Bonferroni–Holm method which reduces the possibility of getting a statistically significant result when performing multiple tests (35).

All lifestyle habits were self-reported, and there may have been problems with misreporting or answering in a perceived socially acceptable manner (recall bias). However, the use of questionnaires is common and has relevance to the clinical setting. The questionnaire in our study had dual purposes, both to evaluate change before and after participating in the structured lifestyle programme, and to enhance awareness of current lifestyle behaviour for the individual.

Another aspect is the ‘Hawthorne effect’, i.e. a change in behaviour of the research participants and/or the health-care provider due to the attention they receive regardless of the intervention (36). This might influence the results of our study, and both this and the aspect of recall bias should be considered interpreting the results.

Our results support the utility of a multifactorial, structured approach in cardiovascular risk prevention for change in unhealthy lifestyle habits in a primary care setting. However, since this study is a single-group study there is a need for future randomized controlled studies to confirm our findings.

## Supplementary Material

Supplemental Material
