# Supplementary material for: Improved unhealthy lifestyle habits in patients with high cardiovascular risk: results from a structured lifestyle programme in primary care
Source: Ups J Med Sci. 2019 May 7;124(2):94–104. doi: 10.1080/03009734.2019.1602088 (PMC6566702; doi:10.1080/03009734.2019.1602088)
Supplement: Supplemental Material [file IUPS_A_1602088_SM8387.docx]

**Welcome to Citypraktiken’s lifestyle unit!**

**You will meet: [name] specialized nurse at …. am/pm, year-month-day, for lifestyle counselling. The appointment will last about 90 minutes.**

**After visiting the nurse you will meet: [name], a physiotherapist, and will perform a bicycle ergometer test. For your convenience, please bring sneakers and a t-shirt. Do not use nicotine or drink coffee or cola within 2 hours before the test.**

**Please check your heart rate before the visit.** (calculate your heart rate for 1 minute before getting out of bed in the morning.)

**If you are unable to come, please make us a call: 021-10 96 80**

**Cost: 100 kr. Please register at reception. Absence will be debited.**

***Please complete the questions on the following pages and bring the leaflet to your appointment.***

**Lifestyle is very important to health and the risk of becoming ill.**

**To help guide you to better health and quality of life, we ask you to complete this questionnaire and bring it with you to your next visit.**

*How do you perceive your health in general?*

□ very good □ good □ not so good □ not at all good

*How would you rate your risk of future cardiovascular illness?*

□ very low □ low □ fairly high □ very high

**PHYSICAL ACTIVITY**

*How physically demanding is your occupation?*

□ very easy □ easy □ strenuous □ very strenuous

*How do you commute to work?*

□ walking □ by bicycle □ by car □ by bus or train

*How physically active are you during the day?*

*(e.g., taking a walk, bicycling, taking the dog for a walk, cleaning the house, gardening, shovelling snow, playing with your children)*

□ not at all physically active □ <30 min per day □ 30–60 min per day □ >60 min per day

*How much exercise do you perform in a week?*

*(e.g., gymnastics, jogging, swimming, ball games, riding)*

□ no activity at all □ <1 hour weekly □ 1–2 hours weekly □ >2 hours weekly

*Please estimate the amount of time you sit each day.*

□ 0–4 hours □ 5–8 hours □ 9–12 hours □ 13 hours or more

*How would you rate your physical fitness?*

□ very good □ good □ not so good □ poor

**DIET**

*How many days a week do you eat breakfast?*

□ every day □ 4–6 days □ 1–3 days □ never

*How many days a week do you eat lunch?*

□ every day □ 4–6 days □ 1–3 days □ never

*How many sandwiches do you eat daily?*

□ ≤2 daily □ 3–4 daily □ 5–6 daily □ >6 daily

*How often do you eat fish?*

□ a couple of times a week or more □ once a week □ a couple of times a month □ rarely/never

*How often do you eat sausages, hamburger or pizza?*

□ a couple of times a week or more □ once a week □ a couple of times a month □ rarely/never

*How often do you eat fruit and vegetables?*

□ daily □ 3–5 times a week □ 1–2 times a week □ a few times a month or less

*How often do you eat “extra” calories?*

□ daily □ 3–5 times a week □ 1–2 times a week □ a few times a month or less

*How often do you drink sweetened soft drinks or juice?*

□ daily □ 3–5 times a week □ 1–2 times a week □ a few times a month or less

**ALCOHOL**

*How often do you drink alcoholic beverages?*

□ never □ less than once a month □ 2–4 times a month □ 1–3 times a week □ ≥4 times a week

*How many “glasses” do you typically drink when you drink alcohol?*

(One glass equals 45 cl light beer, 33 cl beer, 12 cl wine, 8 cl fortified wine, 4 cl spirits)

□ 1–2 glasses □ 3–4 glasses □ 5–6 glasses □ 7–9 glasses □ ≥10 glasses

*Are you or your relatives worried about your current alcohol consumption?*

□ yes □ no

**TOBACCO**

*Do you smoke?*

□ yes □ no

If yes, how many cigarettes daily? …cigarettes

*Do you use snuff?*

□ yes □ no

If yes, how many portions daily? … portions

**STRESS**

*Do you feel stressed?*

□ never □ rarely □ sometimes □ often

*Have you experienced difficulties with sleep?*

□ never □ rarely □ sometimes □ often

*Do you feel anxious?*

□ never □ rarely □ sometimes □ often

*Do you feel depressed?*

□ never □ rarely □ sometimes □ often

*Do you feel tired?*

□ never □ rarely □ sometimes □ often

*Are you affected by any form of body pain?*

□ never □ rarely □ sometimes □ often
